# Decreased susceptibility of *Neisseria gonorrhoeae* isolates from Switzerland to Cefixime and Ceftriaxone: antimicrobial susceptibility data from 1990 and 2000 to 2012

**DOI:** 10.1186/1471-2334-13-603

**Published:** 2013-12-26

**Authors:** Helen Kovari, Maria DG de Melo Oliveira, Paula Hauser, Severin Läuchli, Jürg Meyer, Rainer Weber, Reinhard Zbinden

**Affiliations:** 1 Division of Infectious Diseases and Hospital Epidemiology, University Hospital Zurich, University of Zurich, Zurich, Switzerland; 2 Institute of Medical Microbiology, University of Zurich, Zurich, Switzerland; 3 Division of Dermatology, University Hospital Zurich, University of Zurich, Zurich, Switzerland

**Keywords:** Neisseria gonorrhoeae, Gonorrhoea, Antimicrobial resistance, Cephalosporins

## Abstract

**Background:**

*Neisseria gonorrhoeae* can rapidly develop resistance to antimicrobial agents. Over the last years, decreased gonococcal susceptibility to third-generation cephalosporins, especially cefixime, emerged worldwide. Therefore, current international guidelines recommend dual therapy for gonorrhoea with ceftriaxone plus either azithromycin or doxycycline. Gonococcal susceptibility data in Switzerland are sparse.

**Methods:**

We investigated the prevalence of antibiotic susceptibility of *N. gonorrhoeae* in specimens collected between 1990 and 2012 at the University of Zurich, Switzerland. Minimum inhibitory concentrations (MICs) for cefixime, ceftriaxone, ciprofloxacin, and penicillin were determined by Etests. The European Committee on Antimicrobial Susceptibility Testing (EUCAST) breakpoints were used to define reduced susceptibility.

**Results:**

A total of 320 isolates were tested. Between 1990 and 2006 all tested samples were susceptible to both cephalosporins. Subsequently, the prevalence of elevated MICs for cefixime increased to 10.4% (2007/2008), 11.5% (2009/2010), and 11.4% (2011/2012); and for ceftriaxone to 2.4% (2007/2008), 4.7% (2009/2010), and 0% (2011/2012), respectively. The prevalence of resistance to ciprofloxacin (72.7%) and penicillin (22.7%) was high in 2011/2012.

**Conclusions:**

Decreasing susceptibility of *N. gonorrhoeae* to third-generation cephalosporins in Switzerland supports treatment recommendations with ceftriaxone plus azithromycin or doxycycline. Health-care providers need to be aware of possible treatment failures with cephalosporins. Continued surveillance of gonococcal antimicrobial resistance is essential.

## Background

Infections with *Neisseria gonorrhoeae* may cause serious complications, including pelvic inflammatory disease, infertility, peritonitis, and chronic pain, and it enhances HIV transmission [1,2]. Effective treatment of gonorrhoea is mandatory to control disease transmission and prevent sequelae. However, *N. gonorrhoeae* rapidly developed antimicrobial resistance to all previously recommended first-line drugs, including penicillins, tetracyclines, and fluoroquinolones. Third-generation cephalosporins, such as cefixime and ceftriaxone, remain the only readily available active antimicrobial class. In recent years, however, gonococcal strains with reduced susceptibility to cefixime emerged and spread worldwide [3]. Clinical treatment failures with cefixime have been reported in Japan, several European countries, Canada, and South Africa [4-10]. In 2009, the first treatment failures to ceftriaxone were observed in Australia [11] and 2010 in Sweden [12]. Currently, the identification of extensively drug-resistant (XDR) *N. gonorrhoeae* strains with high-level ceftriaxone resistance in Japan [13], France [14], and Spain [15], are of major concern. In addition to high ceftriaxone minimum inhibitory concentrations (MICs), these XDR strains are typically multidrug-resistant, exhibiting additional resistance to fluoroquinolones as well as to older drugs. Consequently, most treatment guidelines now recommend combination therapy with the injectable agent ceftriaxone plus either azithromycin or doxycycline instead of oral monotherapy with cefixime [16,17].

In Switzerland, a strong rise in *N. gonorrhoeae* infections was observed during the past decade. Notifications have increased nearly threefold, with 521 reported cases in 2003, and 1569 cases in 2012, of which 77% occurred in men [18]. Reporting gonorrhoea to public health authorities has been mandatory in Switzerland since 1988. However, gonococcal susceptibility data in Switzerland are sparse [19]. Due to the current *N. gonorrhoeae* resistance problem, improving gonococcal antimicrobial resistance surveillance is of utmost importance and high on the agenda of the WHO’s “Global action plan to control the spread and impact of antimicrobial resistance in *Neisseria gonorrhoeae*, 2012” [20]. Local antimicrobial surveillance is essential for evidence-based treatment recommendations. Given the lack of data, we investigated the distribution of antibiotic susceptibility of *N. gonorrhoeae* in specimens collected between 1990 and 2012.

## Methods

All isolates were obtained in the northeastern part of Switzerland and the region of Zurich between January 1990 and December 2012. Specimens were cultured from symptomatic gonorrhoea patients with only one isolate taken per patient in case they were infected at multiple sites. Species confirmation and susceptibility testing was performed at the Institute of Medical Microbiology, University of Zurich. Specimens were cultured on selective media, i.e. until 2009 on Thayer Martin agar (Difco; Becton, Dickinson and Company, Basel, Switzerland), supplemented with IsoVitalex, and after 2009 on VCA3 agar (BioMérieux, Marcy-l’Etoile, France). The commercial biochemical gallery Api NH (BioMérieux) was used for the identification.

Minimum inhibitory concentrations (MICs) for cefixime, ceftriaxone, ciprofloxacin and penicillin were determined by the Etest method (BioMérieux, Marcy l’Etoile, France) on Difco GC agar until 2004 (Becton Dickinson, Cockeyswille, MD, USA) and after 2005 on chocolate agar with PolyViteX (BioMérieux) or on MH-horse blood agar (BioMérieux). In order to demonstrate the comparability of the different methods, we tested 9 isolates (1990–2004) and the reference ATCC strain 49226 with all three media for all four antibiotics (Additional file 1: Table S1). Beta-lactamase test was performed with nitrocefin (Becton Dickinson). The European Committee on Antimicrobial Susceptibility Testing (EUCAST) breakpoints were used [21]: Breakpoints for the definition of decreased cefixime and ceftriaxone susceptibility were MICs >0.125 mg/L, and ciprofloxacin resistance was defined by MICs >0.064 mg/L. Penicillin resistance was defined by either MICs >1.0 mg/L or the presence of penicillinase-producing strains.

The following data was available for all isolates collected between 2005 and 2012: specimen source, date specimen obtained, sex, age, and specimen site. For all isolates collected before 2005 only date of specimen collection was available. The study was approved by the ethics committee of Zurich (ethics approval number: KEK-ZH-Nr. 2012–0510).

## Results

A total of 318 isolates from the region of Zurich and the northeastern part of Switzerland were analysed, including 42 samples from 1990, 66 from 2000 to 2004, and 210 samples from 2005 to 2012. The level of coverage (number of isolates tested compared to the number of reported cases in the region of Zurich) was 5%. Of the samples collected between 2005 and 2012, 168 (79.3%) were collected from men, 35 (16.5%) from women, and in 9 (4.2%) samples data on sex and age was missing. Median age for males was 35 years (range 17 to 86 years) and for females 31 years (range 16 to 66 years). The isolates were obtained from the urethra (60%), cervix (13%), other locations (4%), including anorectum, pharynx, joint and eye, or from unknown sites (24%).

**Figure 1:**
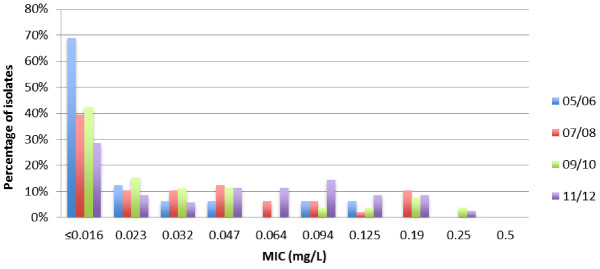
**Distribution of minimum inhibitory concentrations of *Neisseria gonorrhoea *isolates for cefixime, 2005–2012.** 2005/2006: 17 isolates. 2007/2008: 48 isolates. 2009/2010: 26 isolates. 2011/2012: 35 isolates. Abbreviation: MIC, minimum inhibitory concentration.

The results of antimicrobial susceptibilities are summarized in Table 1. No decreased susceptibility to cefixime was detected until 2006 in the tested strains, whereas 2007/2008 10.4%, 2009/2010 11.5%, and 2011/2012 11.4% of isolates displayed in vitro resistance to cefixime. In Figure 1 cefixime MIC distribution between 2005 and 2012 is illustrated. From 2005 onwards, isolates demonstrate a clear shift towards higher MIC categories.

**Table 1:** ***N. gonorrhoeae *** with decreased susceptibility* to cefixime, ceftriaxone, and resistance to ciprofloxacin and penicillin in Switzerland, 1990–2012

	**Cefixime**			**Ceftriaxone**			**Ciprofloxacin**			**Penicillin**		
**Year**	**Tested, n**^**#**^	**DS, n (%)**	**(95% CI)**	**Tested, n**^**#**^	**DS, n (%)**	**(95% CI)**	**Tested, n**^**#**^	**R, n (%)**	**(95% CI)**	**Tested, n**^**#**^	**R, n (%)**	**(95% CI)**
**1990**	16	0 (0)	(0–21)	16	0 (0)	(0–21)	42	1 (2.4)	(0–12)	42	9 (21.4)	(10–37)
**2000-2002**	7	0 (0)	(0–40)	7	0 (0)	(0–40)	33	10 (30.3)	(15–48)	33	4 (12.1)	(3–28)
**2003/2004**	10	0 (0)	(0–30)	10	0 (0)	(0–30)	33	16 (48.5)	(31–66)	33	13 (39.4)	(23–58)
**2005/2006**	17	0 (0)	(0–19)	32	0 (0)	(0–11)	36	21 (58.3)	(41–74)	31	7 (22.6)	(10–41)
**2007/2008**	48	5 (10.4)	(3.4-22)	85	2 (2.4)	(0.3-8)	85	62 (72.9)	(62–82)	84	25 (29.8)	(20–41)
**2009/2010**	26	3 (11.5)	(2.4-30)	43	2 (4.7)	(0.5-16)	43	31 (72.1)	(56–84)	43	7 (16.3)	(7–30)
**2011/2012**	35	4 (11.4)	(3.2-26)	46	0 (0)	(0–8)	44	32 (72.7)	(57–85)	44	10 (22.7)	(11–38)
**Total**	159			239			317			311		

*Abbreviations*: CI, confidence interval of the proportion; DS, decreased susceptibility; R, resistant.

*Decreased susceptibility to cefixime and ceftriaxone defined by minimal inhibitory concentration (MIC) >0.125 mg/L. Ciprofloxacin resistance defined by MIC >0.064 mg/L, and penicillin resistance defined by MIC >1.0/L or penicillinase-producing strains, according to the European Committee on Antimicrobial Susceptibility Testing (EUCAST).

^#^The 42 isolates from 1990 and the 66 isolates from 2000–2004 were stored at -70°C and subcultured in 2005 for testing for penicillin and ciprofloxacin; and in 2011 for cefixime and ceftriaxone. The 36 strains from 2005/2006, the 85 strains from 2007/2008, the 43 strains from 2009/2010, and the 44 strains from 2011/2012 were tested under routine conditions for penicillin, ciprofloxacin and ceftriaxone. In 2011 the viable strains were tested for cefixime. By the end of 2011, all strains were tested routinely for penicillin, ciprofloxacin, ceftriaxone and cefepime. This explains why the number of strains tested in each period differs for each antibiotic.

**Figure 2:**
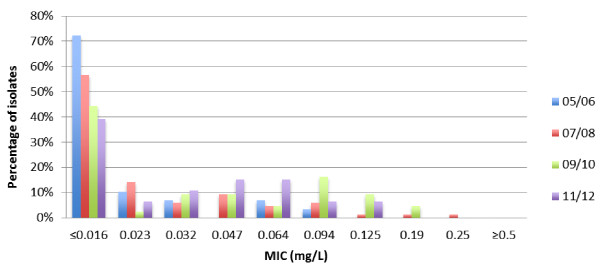
**Distribution of minimum inhibitory concentrations of *Neisseria gonorrhoea *isolates for ceftriaxone, 2005–2012.** 2005/2006: 29 isolates. 2007/2008: 85 isolates. 2009/2010: 43 isolates. 2011/2012: 46 isolates. Abbreviation: MIC, minimum inhibitory concentration.

Ceftriaxone susceptibility was 100% until 2007. Between 2008 and 2010 four isolates exhibited decreased susceptibility to ceftriaxone, with one isolate from 2008 showing a ceftriaxone MIC of 0.25 mg/L. In three of these patients the site of infection was urethral and in one patient anorectal. Most strains showed low ceftriaxone MIC’s of ≤0.016 mg/L between 2005 and 2012. However, the proportion of isolates in higher MIC categories has clearly increased in recent years (Figure 2).

Fluoroquinolone-resistant *N. gonorrhoeae* emerged in Switzerland during the 1990s, with a ciprofloxacin resistance rate of 2.4% in 1990. In the early 2000s, a rapid increase of ciprofloxacin resistance from 30.0% in 2001/2002 to 72.7% in 2011/2012 was observed (Table 1). Penicillin resistance rate was 22.7% in 2011/2012. However, only 18.2% of the strains were fully penicillin susceptible according to EUCAST breakpoints (≤0.06 mg/L).

## Discussion

In Switzerland, *N. gonorrhoeae* strains with decreased susceptibility to third-generation cephalosporins are emerging. We observed increasing rates of cefixime resistant strains, with a prevalence of 11% between 2007 and 2012. Moreover, ceftriaxone MICs are rising. The proportion of isolates with elevated ceftriaxone MIC was 2.2% between 2007 and 2012.

Our findings are in line with the worldwide documented decrease in cephalosporin susceptibility of *N. gonorrhoeae*. In the European Gonococcal antimicrobial susceptibility surveillance project (Euro-GASP), 9% of isolates displayed decreased susceptibility to cefixime (MIC ≥0.25 mg/L) in 2010. Cefixime resistance rates were >5% in 11 of 21 European countries, and >15% in 5 countries, including Austria, Denmark, Slovenia, Spain and Cyprus [22]. In the United States the percentage of cefixime resistant isolates (MIC ≥0.25 mg/L) increased from 0.1% in 2006 to 1.4% in 2011 [23]. The rise was most pronounced in the West with resistance rates of 17% in Hawaii and 6.4% in San Diego, California, in 2011 [17].

These are in vitro data and clinical cephalosporin resistance has not yet been defined due to the fact that so far there have been too few documented treatment failures in order to establish a clear relationship between MIC and clinical treatment failure. In a recently published Canadian cohort study the rate of clinical treatment failure associated with isolates of *N. gonorrhoeae* with a cefixime MIC of ≥0.12 mg/L was 25.0% compared to 1.9% of isolates with a cefixime MIC of <0.12 mg/L [9].

In 2008, we isolated one strain with a MIC for ceftriaxone of 0.25 mg/L. No decreased susceptibility to ceftriaxone (MIC ≥0.25 mg/L) was detected from 2004 to 2010 among Euro-GASP isolates. However, in correspondence to our data, ceftriaxone MICs increased from 2009 onwards [22]. In the United States the percentage of isolates with elevated ceftriaxone MICs (≥0.125 mg/L) rose slightly from 0% in 2006 to 0.4% in 2011 [17]. As displayed for cefixime, no threshold for resistance to ceftriaxone in *N. gonorrhoeae* has been defined yet. Increasing MICs for cephalosporins, however, may precede the emergence of resistance.

We found high ciprofloxacin resistance rates of *N. gonorrhoeae*, with 72.7% in 2012. Our data demonstrates that during the 1990s and early 2000s, when ciprofloxacin was first choice for treatment, fluoroquinolone-resistant isolates emerged, and rapidly increased after 2000, correlating with the observation in other countries around the world [24-26]. By 2007, in the United States, and even earlier in other regions, including Europe and Australia, fluoroquinolones were no longer recommended for the treatment of gonococcal infections [27,28]. In Europe, ciprofloxacin resistance was 53% (MIC ≥1 mg/L) in 2010 [22], and 13.3% in the United States in 2011 (≥1 mg/L) [23].

The World Health Organization (WHO) recommends discontinuation of empirical use of an antibiotic once 5% of locally acquired gonococcal isolates are resistant [20]. The high rates of strains with decreased cefixime susceptibility and first isolates with elevated MICs for ceftriaxone shown by our data, support the recommendation of dual therapy for gonococcal infections with ceftriaxone plus either azithromycin or doxycycline. Combination regimens have been adopted as strategies for the treatment of other bacterial infections in the context of multidrug resistance development. The oral cephalosporin, cefixime, should no longer be used as first-line treatment.

In light of identified strains with decreased ceftriaxone susceptibility, health-care providers need to be vigilant for treatment failures, even in patients treated with recommended antibiotic regimens. In persons treated with alternative regimens, such as cefixime, test of cure is recommended. Continued local surveillance of gonococcal antimicrobial susceptibility is important. Due to the widespread use of nucleic acid amplification testing, culture for *N. gonorrhoeae* with antimicrobial susceptibility testing is not routinely done in clinical practice.

To the best of our knowledge, this is the first report on cephalosporin susceptibility testing of *N. gonorrhoeae* in Switzerland. Longitudinal data on antimicrobial resistance in gonococci over a period of more than two decades was assessed. Nevertheless, the study has several limitations. The level of coverage was relatively low, attributed in a large part to the low number of gonococcal cultures taken routinely in clinical practice. The samples were not systematically collected within a sentinella system, which may result in selection of specimens with a higher resistance rate. Not all strains were tested for cephalosporins, which particularly concerns isolates obtained before 2006. However, the subset of 20-30% of all isolates stored, none showed decreased susceptibility for cefixime and ceftriaxone. It is therefore unlikely that we missed a relevant resistance signal. Finally, the isolates were not tested for azithromycin.

## Conclusions

We found a high prevalence of *N. gonorrhoeae* with decreased susceptibility to cefixime, first strains with elevated MICs for ceftriaxone, and high resistance rates to ciprofloxacin. Treatment of gonorrhea with a combination regimen with ceftriaxone plus azithromycin or doxycycline is supported. Our data contributes to the global antimicrobial resistance surveillance of *N. gonorrhoeae,* and emphasizes the importance of further gonococcal antimicrobial resistance testing.

## Competing interests

The authors declare that they have no competing interests.

## Authors’ contributions

HK, RZ and RW designed the study. SL and JM provided gonococcal isolates. MDM and RZ performed the laboratory analyses. All authors analysed and interpreted the data. HK and PH prepared the paper. All authors read and approved the final manuscript.

## Pre-publication history

The pre-publication history for this paper can be accessed here:

http://www.biomedcentral.com/1471-2334/13/603/prepub

## Supplementary Material

Additional file 1: Table S1. Minimal inhibitory concentrations for 9 *Neisseria gonorrhoeae* isolates (1990 – 2004) and for the reference ATCC strain 49226 for penicillin, ceftriaxone, cefixime and ciprofloxacin on chocolate agar with PolyViteX (BioMérieux), Difco GC agar (Becton Dickinson, Cockeyswille, MD, USA), and on MH-horse blood agar (BioMérieux) showing little variation. Click here for file
