# Supplementary material for: Decreased susceptibility of Neisseria gonorrhoeae isolates from Switzerland to Cefixime and Ceftriaxone: antimicrobial susceptibility data from 1990 and 2000 to 2012
Source: BMC Infect Dis. 2013 Dec 26;13:603. doi: 10.1186/1471-2334-13-603 (PMC3881505; doi:10.1186/1471-2334-13-603)
Supplement: Additional file 1: Table S1. — Minimal inhibitory concentrations for 9 Neisseria gonorrhoeae isolates (1990 – 2004) and for the reference ATCC strain 49226 for penicillin, ceftriaxone, cefixime and ciprofloxacin on chocolate agar with PolyViteX (BioMérieux), Difco GC agar (Becton Dickinson, Cockeyswille, MD, USA), and on MH-horse blood agar (BioMérieux) showing little variation. [file 1471-2334-13-603-S1.doc]

**Additional file 1**

**Table S1**: Minimal inhibitory concentrations for 9 *Neisseria gonorrhoeae* isolates (1990 – 2004) and for the reference ATCC strain 49226 for penicillin, ceftriaxone, cefixime and ciprofloxacin on chocolate agar with PolyViteX (BioMérieux), Difco GC agar (Becton Dickinson, Cockeyswille, MD, USA), and on MH-horse blood agar (BioMérieux) showing little variation.

|  | **Penicillin MIC (mg/L)** | | | **Ceftriaxone MIC (mg/L)** | | | **Cefixime MIC (mg/L)** | | | **Ciprofloxacin MIC (mg/L)** | | |
| --- | --- | --- | --- | --- | --- | --- | --- | --- | --- | --- | --- | --- |
| **N.gonorrhoeae isolates** | **PVX** | **Difco GC agar** | **MHF** | **PVX** | **Difco GC agar** | **MHF** | **PVX** | **Difco GC agar** | **MHF** | **PVX** | **Difco GC agar** | **MHF** |
| **ATCC 49226** | 0.5 | 0.5 | 0.38 | 0.016 | 0.016 | <0.016 | 0.047 | 0.032 | 0.023 | 0.006 | 0.004 | 0.004 |
| **Strain 1** | 0.38 | 0.38 | 0.5 | <0.016 | <0.016 | <0.016 | <0.016 | 0.023 | <0.016 | 0.023 | 0.023 | 0.016 |
| **Strain 2** | 0.25 | 0.38 | 0.38 | <0.016 | <0.016 | <0.016 | <0.016 | <0.016 | <0.016 | 0.016 | 0.012 | 0.008 |
| **Strain 3** | 0.75 | 1 | 0.75 | <0.016 | <0.016 | <0.016 | 0.023 | 0.023 | 0.016 | 0.023 | 0.023 | 0.023 |
| **Strain 4** | 0.094 | 0.125 | 0.047 | <0.016 | <0.016 | < 0.016 | <0.016 | <0.016 | <0.016 | 0.006 | 0.004 | 0.002 |
| **Strain 5** | 1 | 0.75 | 0.5 | 0.047 | 0.047 | 0.032 | 0.125 | 0.064 | 0.064 | 0.19 | 0.19 | 0.25 |
| **Strain 6** | >32 | >32 | >32 | <0.016 | <0.016 | < 0.016 | <0.016 | <0.016 | <0.016 | 1.5 | 1.5 | 1.5 |
| **Strain 7** | >32 | >32 | >32 | <0.016 | 0.016 | < 0.016 | 0.023 | 0.023 | 0.023 | >32 | >32 | >32 |
| **Strain 8** | >32 | >32 | >32 | 0.023 | 0.032 | 0.023 | 0.047 | 0.047 | 0.047 | >32 | >32 | >32 |
| **Strain 9** | 16 | 12 | 8 | <0.016 | <0.016 | <0.016 | 0.023 | 0.023 | 0.047 | 0.008 | 0.008 | 0.008 |

Abbreviations: MHF, MH-horse blood agar; MIC, minimal inhibitory concentrations; PVX, chocolate agar with PolyViteX (BioMérieux).
